# *Klebsiella pneumoniae*-induced liver and prostate abscesses

**DOI:** 10.1590/0037-8682-0262-2023

**Published:** 2023-07-24

**Authors:** Chee Yik Chang

**Affiliations:** 1 Medical Department, Hospital Sungai Buloh, Selangor, Malaysia. Medical Department Hospital Sungai Buloh Selangor Malaysia

**FIGURE 1:**
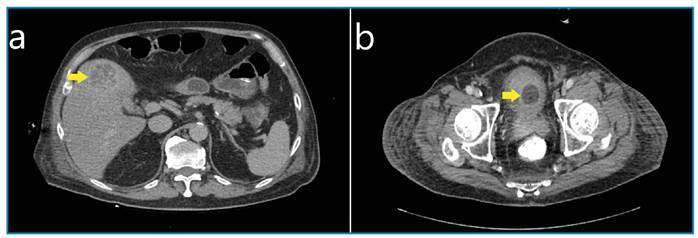
A CT scan of the abdomen and pelvis showing **(a)** a liver abscess in segment V measuring 3.9 x 3.6 x 2.9 cm and **(b)** a prostate abscess measuring 3.5 x 3.2 x 3.7 cm.

A 72-year-old man with diabetes mellitus presented with a 2-day history of fever, lethargy, and shortness of breath. Upon admission, the patient was in a septic shock with severe respiratory distress, which required the administration of inotropic agents and mechanical ventilation. An urgent computed tomography (CT) scan of the abdomen and pelvis revealed a liver abscess in segment V and a prostate abscess (Figure 1). No physical examination findings indicated meningitis or endophthalmitis. He was administered an empirical antibiotic (intravenous piperacillin-tazobactam). Blood cultures revealed *Klebsiella pneumoniae*, which was susceptible to ampicillin-sulbactam. Consequently, we switched to ampicillin-sulbactam. However, the patient's clinical condition deteriorated, and he developed severe thrombocytopenia due to sepsis, which precluded surgical drainage. A 1-week follow-up scan revealed that the size of the liver and prostate abscesses had reduced slightly. Unfortunately, the patient passed away 3 weeks later because of a nosocomial infection.

Compared with classic *K. pneumoniae* strains, hypervirulent strains of *Klebsiella pneumoniae* are more likely to cause severe disseminated infections such as community-acquired infections, including liver abscesses, pneumonia, meningitis, and endophthalmitis^1^. Prostate abscess is a rare complication of *K. pneumoniae* infection; however, in Taiwan, *K. pneumoniae* is the most common pathogen associated with prostate abscess, especially in patients with diabetes^2^. The optimal management of *K. pneumoniae*-induced liver and prostate abscesses includes confirming via imaging, administering appropriate antimicrobial therapy, and ensuring adequate drainage^3^.
